# Molecular targets and oxidative stress biomarkers in hepatocellular carcinoma: an overview

**DOI:** 10.1186/1479-5876-9-171

**Published:** 2011-10-10

**Authors:** Monica Marra, Ignazio M Sordelli, Angela Lombardi, Monica Lamberti, Luciano Tarantino, Aldo Giudice, Paola Stiuso, Alberto Abbruzzese, Rossella Sperlongano, Marina Accardo, Massimo Agresti, Michele Caraglia, Pasquale Sperlongano

**Affiliations:** 1 Department of Biochemistry and Biophysics, Second University of Naples, Naples, Italy; 2 Department of Anaesthesiology and Special Surgery, Second University of Naples, Naples, Italy; 3 Departement of Experimental Medicine, Sezione di Medicina del lavoro, Igiene e Tossicologia Industriale, Second University of Naples, Naples, Italy; 4 Interventional US Unit, Department of Medicine, S. Giovanni di Dio Hospital, 80059 Torre del Greco (Naples), Italy; 5 Animal Facility Unit, National Institute of Tumours "Fondazione G. Pascale" of Naples, Naples, Italy; 6 Department of Morphopathology, II University Naples, Napoli, Italy

## Abstract

Hepatocellular carcinoma (HCC) is a complex and heterogeneous tumor with multiple genetic aberrations. Several molecular pathways involved in the regulation of proliferation and cell death are implicated in the hepatocarcinogenesis. The major etiological factors for HCC are both hepatitis B virus (HBV) and hepatitis C virus infection (HCV).

Continuous oxidative stress, which results from the generation of reactive oxygen species (ROS) by environmental factors or cellular mitochondrial dysfunction, has recently been associated with hepatocarcinogenesis. On the other hand, a distinctive pathological hallmark of HCC is a dramatic down-regulation of oxido-reductive enzymes that constitute the most important free radical scavenger systems represented by catalase, superoxide dismutase and glutathione peroxidase.

The multikinase inhibitor sorafenib represents the most promising target agent that has undergone extensive investigation up to phase III clinical trials in patients with advanced HCC. The combination with other target-based agents could potentiate the clinical benefits obtained by sorafenib alone. In fact, a phase II multicenter study has demonstrated that the combination between sorafenib and octreotide LAR (So.LAR protocol) was active and well tolerated in advanced HCC patients.

The detection of molecular factors predictive of response to anti-cancer agents such as sorafenib and the identification of mechanisms of resistance to anti-cancer agents may probably represent the direction to improve the treatment of HCC.

## Introduction

Hepatocellular carcinoma (HCC) is the most common type of primary liver cancer representing the 85% of liver cancers. Other types of liver cancer include cholangiocarcinoma, which starts in the cells that line the bile duct, angiosarcoma (or haemangiosarcoma), which starts in the blood vessels of the liver, and hepatoblastoma which is very rare and usually affects young children.

HCC accounts for up to 75% to 85% of primary liver cancer in the United States (U.S.) [1] and for over 90% in high-risk areas. It predominantly affect people in developing countries, such as sub-Saharan Africa, China, Taiwan, Korea, or Vietnam [2,3].

The incidence has been increasing in recent years in the Mediterranean countries, including Italy, where the incidence and mortality rates are at a median frequency compared to other populations, and it represents the seventh cause of death for tumor, with about 5,000 deaths per year [4-6].

Liver cirrhosis is present in about 90% of HCC [7] mainly caused by chronic infection by hepatitis B (HBV) and C (HCV) viruses [2,8-12] and/or alcohol assumption.

Race, heavy alcohol use, cigarette smoking, obesity, and mellitus diabetes have also been associated with an increased risk of developing HCC. HCC is now more often associated with HCV, particularly in developed countries. On the other hand, HCC is now decreasing in HBV endemic countries due to the implementation of vaccination programs while it is increasing in cohorts who have been infected with chronic HCV [13-22].

## 1. Hepatocarcinogenesis

The molecular mechanism of hepatocarcinogenesis is very intricated. Cancer cells have defects in regulatory genes that govern normal cell proliferation and homeostasis due to a progressive accumulation of mutations. The alterations in cell physiology that collectively dictate malignant growth are: i) self-sufficiency in growth signals (activation of oncogenes); ii) insensitivity to growth-inhibitory signals (inactivation of anti-oncogenes or tumor suppressor genes); iii) escape from apoptosis; iv) limitless replicative potential; v) neo-angiogenesis and tissue invasion and metastases [23].

In fact, hepatocarcinogenesis is considered a multistep process involving subsequent mutations of genes that control proliferation and/or apoptosis in the hepatocytes subjected to continuous inflammatory and regenerative stimuli, starting from the initial phases of chronic hepatitis and then of liver cirrhosis.

HCC is associated with, and preceded by, a number of morphologically distinct lesions. The latter are collectively described as 'preneoplastic lesions', and include dysplastic foci and dysplastic nodules. Hepatic nodules in patients with chronic liver diseases are subdivided into regenerative nodules (mono acinus and multi acinus), low-grade dysplastic nodules, high-grade dysplastic nodules, well-differentiated HCC, moderately-differentiated HCC, and poorly-differentiated HCC, in an ascending order of histologic grades, representing a sequence of multistep hepatocarcinogenesis. Accumulation of genetic alterations in the preneoplastic lesions is believed to lead to the development of HCC. Genomic alterations occur randomly, and they accumulate in dysplastic hepatocytes and HCC. Although genetic changes may occur independently of etiologic conditions, some molecular mechanisms have been more frequently related to a specific etiology [24-26].

Under normal physiological conditions, hepatocyte turnover is very low with a half-life estimated at 6 months. However, adult liver cells retain the remarkable capacity to proliferate in response to injury or to the loss of liver mass. Progenitor cells (also referred to as oval cells) do not play a major role in this growth response but, the same 'resting' differentiated hepatocytes re-enter the cell cycle and replicate once or twice during the period of mass restoration before returning to a state of quiescence. In about 40% of HCC, progenitor cells express peculiar bio-markers (CK-7, CK-19, CD34) associated with a poor prognosis and with disease recurrence [27].

### 1.1 Role of HBV and HCV viruses

HBV and HCV viruses can be implicated in the development of HCC in an indirect way, through induction of chronic inflammation, or directly by means of viral proteins or, in the case of HBV, by creation of mutations by integration into the genome of the hepatocyte.

On HCV-infected patients the development of HCC requires about 10 years from the diagnosis of cirrhosis and about 30 years from exposure to HCV [28]. Conversely, the time course of HBV-related carcinogenesis is less predictable since HCC may precede the occurrence of cirrhosis, particularly with chronic HBV infection in endemic areas [29]

During the 'preneoplastic' phase (chronic hepatitis and cirrhosis), genetic alterations are almost entirely 'quantitative', occurring by epigenetic mechanisms without changes in the structure of genes. In this phase, hepatocytes undergo an intense mitogenic stimulation due to exposure to elevated levels of growth factors, such as insulin-like growth factor (insulin-like growth factor-2, IGF-2), transforming growth factor-α (TGF-α), interleukin 6 as well as inflammatory cytokines, which may lead to activation of the major signaling pathways involved in cell proliferation and angiogenesis. The enhanced expression of growth factors and cytokines is driven by inflammation, the action of viral proteins and regenerative response to cell loss. The mechanisms whereby these factors affect gene expression include DNA mutations with consequent activation or inactivation of gene promoters [26].

**Figure 1:**
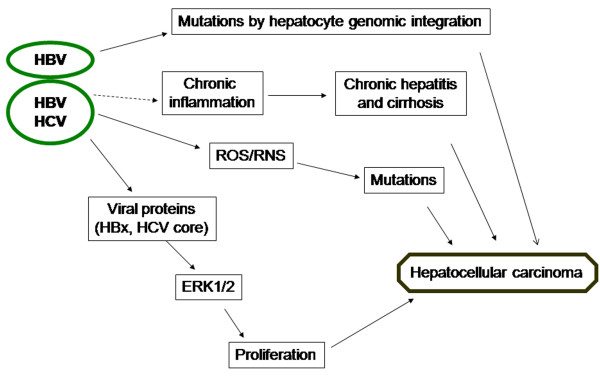
**Key steps in the development of HCC caused by HBV/HCV infection**.

Development of human HCC by viral (HBV and HCV) factors is resumed in Figure 1.

#### HBV virus

HBV belongs to a family of closely related DNA viruses, called Hepadnaviridae [30]. It specifies a small number of known gene products, including a reverse transcriptase/DNA polymerase (pol), capsid protein (core), envelope (env) proteins (L, M and S) as well as proteins of uncertain function such as 'X' and 'e'. It is classified as para-retrovirus because its replication depends upon reverse transcription of genome-length RNA.

The molecular etiology of HBV-induced HCC remains for the most part unclear. However, the viral protein X (HBx) derived by HBV, can directly stimulate the intracellular kinase cascades involved in the regulation of cell proliferation [26,31]. In some HBV-induced HCCs, HBx can inactivate the cellular antioncogene product, p53, which is frequently disabled in HCC [32].

Usually, HBx functions as a transcriptional transactivator of different host genes involved in cellular growth control. HBx transactivates cellular genes involved in cell proliferation control (c-jun, c-fos, c-myc) and growth factor receptors, such as EGF-R, involved in the regulation of cell proliferation and transformation [33]. This transactivation activity appears to involve stimulation of different transcription factors such as CREB (cAMP Responsive Element Binding protein), NFkB (Nuclear Factor kB), AP1 (Activating Protein 1) and NF-AT (Nuclear Factor of Activated T Cells) [34,35].

HBV can transform hepatocytes even in the absence of chronic inflammation and cirrhosis, while the role and significance of the inflammation is more important in the development of HCV-associated HCCs. On the other hand, many transcription- and signalling-related genes were upregulated in HBV-HCCs without cirrhosis. The IGF signal pathway seems to play a central role in HBV-HCCs, especially when developing from a noncirrhotic liver. A higher number of genes were differently expressed between HBV-HCCs associated or not with cirrhosis.

HBV replication appears to involve heat shock proteins [36] and viral envelope gene transcription may be actually upregulated by endoplasmic reticulum (ER stress) which interrupts protein folding causing accumulation of unfolded or misfolded proteins in ER leading to a cell response that involves mutagenic reactions [37]. Hepatitis B virus X protein (HBx) activates ATF6 and IRE1-XBP1 pathways of unfolded protein response [38].

#### HCV virus

Hepatitis C virus is a member of the Flaviviridae family of enveloped, positive-strand RNA viruses [39]. Similar to HBV, HCV utilizes the ER as the primary site of genomic replication and virion assembly [40,41]. Upon entry and uncoating, the RNA viral genome is translated by ER bound ribosomes into a polyprotein that is cleaved by cellular and viral proteases to generate 10 mature viral gene products, including the core protein that forms the viral capsid, NS3, which has the protease and helicase activity, NS5A, and the viral RNA polymerase NS5B. In addition to the proteins derived from the polyprotein coding sequence, the HCV RNA codes for another protein termed the F protein or the alternative reading frame protein (ARFP) using an open reading frame that overlaps with the core protein coding sequence [42,43].

The HCV capside core is a multifunctional protein with regulatory functions that affects transcription and cell growth in vitro and in vivo [44].

The HCV core binds to the p53, p73 and pRb tumor suppressor proteins [45-48], but the functional consequences of these interactions have not fully been elucidated. Hepatitis C virus core protein also modulates the expression of the cyclin-dependent kinase (CDK) inhibitor p21/Waf [49]. Hepatitis C virus core protein is produced as an innate form (amino acids 1-191) that is then processed to produce a mature form (amino acids 1-173). The innate core protein in the cytoplasm increases the amount of p21WAF1 by activating p53, and the mature core protein in the nucleus decreases the amount of p21WAF1 by a p53-independent pathway [50,51].

The ability of HCV core proteins to directly activate the MAP kinase cascade and to prolong its activity in response to mitogenic stimuli may contribute to the neoplastic transformation of HCV infected liver cells [44]. Recently, it was demonstrated that HCV-infection causes ER-stress, Ca2+ homeostasis deregulation and reactive oxygen species (ROS) production by mitochondria that would lead to apoptosis [52-55].

The pathological alterations caused by HCV are similar to the HBV-related disease; acute and chronic hepatitis, cirrhosis and HCC. HCV is not considered as a directly cytotoxic virus; hepatitis occurs as a result of the reaction of the host immune system against the virus infected cells.

Low number of genes were expressed differently between HCV-HCCs associated with and without cirrhosis.

The most effective tool to prevent HCC is avoidance of the risk factors such as viral infection. An effective vaccine has been available for prevention of new infection with HBV; however, no vaccine exists against HCV infection.

## 2. Molecular biomarkers of HCC pathogenesis

The carcinogenesis and progression of HCC is a complex multistep process that involves multiple genetic aberrations. The molecular mechanisms involved in development and progression of HCC are still largely unknown. On the other hand, different molecular markers have been considered as prognostic factors for HCC. To deepen the molecular mechanisms underlying HCC carcinogenesis and progression is important for improving prognosis and treatment strategies.

**Figure 2:**
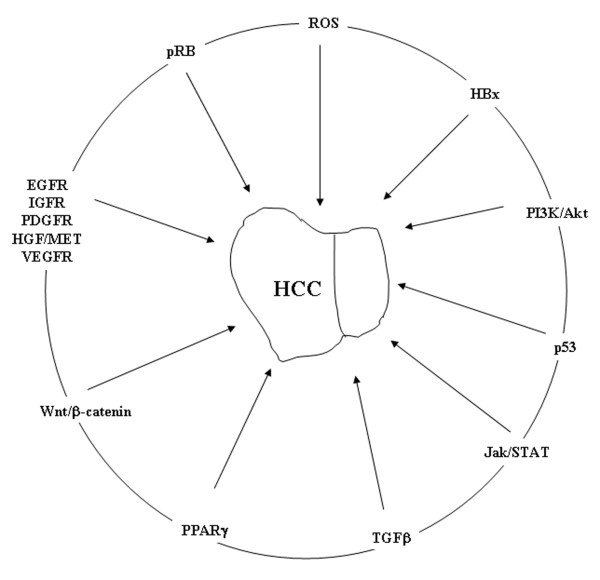
**Cellular signaling pathways implicated in HCC**.

Several molecular pathways involved in the regulation of proliferation and cell death are implicated in the hepatocarcinogenesis (Figure 2). In fact, experimental studies have shown structural genomic changes in very early stages of hepatocarcinogenesis. Genomic instability, rearrangements and transactivation of Ras and β-catenin signaling are induced by the integration of HBV into hepatocyte genome [26,56]. HCV core protein also upregulates TGF-α and IGF-2 [57-60].

The most common genetic alterations in HCC can be grouped into 3 main routes: i) p53- ii) Wnt- and iii) RB1-dependent pathways [61]

The binding of Wnt proteins to specific Frizzled receptors on the surface of target cells activates distinct intracellular pathways. This results in the accumulation and nuclear localization of the β-catenin protein characteristic of canonical Wnt pathway activation that targets specific genes including cyclin D1, c-Myc, and survivin, which are critical for cancer development [62,63]. In fact, a transgenic mice model suggested that high expression of Wnt-1 could be the major cause for nuclear accumulation of β-catenin, which subsequently contributes to c-myc/E2F1-driven hepatocarcinogenesis [64]. Clinical studies have reported that abnormal activation of Wnt/β-catenin pathway is frequently involved in hepatocarcinogenesis. About 33-67% of HCC tissues show accumulation of β-catenin in the cytoplasm and nucleus, whereas no accumulation was observed in the corresponding normal tissues [65,66]. In addition, upregulation of upstream elements such as Frizzled receptors was reported to be involved in HCC development and progression [67,68]. The activation of Wnt/β-catenin signaling was abolished by a knockdown of Frizzled-7 receptor expression by siRNA. More important, a specific Wnt3-Frizzled-7 receptor interaction was observed by co-immunoprecipitation experiments, which suggest that the action of Wnt3 was mediated via Frizzled-7 receptor [69].

In HCC, proteomics results suggested that enhanced Wnt-1 expression associated with NF-kB might be an important mechanism underlying hepatocarcinogenesis [70].

MAPK cascade transduces signals from tyrosine kinase receptors, such as EGFR, IGFR, Platelet-derived growth factor receptor (PDGFR), Hepatocyte growth factor receptor (HGF/MET), and Vascular endothelial growth factor receptor (VEGFR). In this cascade, active Ras (Ras-GTP) triggers the sequential activation of RAF-1, MEK-1/2, and ERK-1/2. The activation/phosphorylation of ERK1/2 allow to enter into the nucleus where transactivates numerous growth-related genes, including c-JUN, c-FOS, c-MYC (involved in the proliferation and survival mechanisms), vascular endothelial growth factor (VEGF) and hypoxia-induced factor (HIF-1α) that regulates angiogenesis, and HKII (Hexokinase II) [71-73]. The constitutive activation of ERK1/2 can determine an increase of cell proliferation also in absence of growth factor. This condition can lead to tumour progression.

Genes that are components of MAPK cascade, such as Ras-GTP, c-RAF, c-FOS, and c-JUN, may be upregulated in HCC induced in rodents [58,74]. 3-Hydroxy-3-methylglutaryl-CoA reductase gene, encoding a key enzyme for de novo synthesis of mevalonate, a precursor of isoprenoid residues necessary for activation of Ras, is upregulated in rat and human liver lesions [75].

Recent studies have shown high levels of active Ras, accompanied by modest/no increase in active RAF-1 and pMEK-1/2, in HCC. This is compatible with the strong induction of the inhibitors of phosphorylation/activation of RAF-1 and MEK-1/2: disabled homolog 2 (Dab2), and RAF kinase inhibitory protein (RKIP), respectively [73].

Up-regulation of principal mediators of the pathway, H-ras and B-RAF, was detected in HCC confirming their role in cancer. Different mechanisms account for Ras signaling in HCC, including:

i) H-ras overexpression; ii) DNA copy number gains in B-RAF genomic locus (chromosome 7q34); iii) epigenetic mechanisms involving the methylation of tumor suppressor genes RASSF1A and NORE1A [76].

The Ras-RAF-ERK-dependent pathway is implicated in the molecular pathogenesis of HCC for three reasons: i) Ras protein is activated in the 30% of cases of HCC [77]; ii) the over-expression of Raf kinase is in the majority of HCC [78]; iii) several upstream growth factors, such as EGF, VEGF, PDGF, TGFα, generally over-expressed in HCC, can activate this pathway binding proper tyrosin kinase receptors [79].

Recently developed technology, such as DNA microarrays and other molecular profiling techniques, has provided new insights into the molecular genetics of HCC [80,12].

HCC are classified in metabolic pathways, and the most represented are the Aryl Hydrocarbon receptor signalling (AHR), involved in the activation of the cytosolic aryl hydrocarbon receptor by structurally diverse xenobiotic ligands (including dioxin, and polycyclic or halogenated aromatic hydrocarbons) and mediating their toxic and carcinogenic effects [81] and, protein Ubiquitination pathways, involved in cell-cycle regulation as well as cell death/apoptosis [82] through modification of target proteins.

Moreover, molecular profiling has been successfully used to identify candidate genes for HCC such as genes correlated with tumour progression (p16, SOCS1, PEG10), metastatization (NM23-H1, osteopontin, RhoC, KAI1, MMP14) or recurrence (REL, A20, vimentin, PDGFRA) [83].

## 3. Involvement of Oxidative stress in HCC

Studies of mechanisms of oxidative stress have shown that it activates signaling cascades (including MAPK pathway), which can seriously influence regulation of cell growth and transformation processes [84]. Particularly, MAP kinases may be involved in pathogenesis of some diseases associated with oxidative stress.

It is known that the oxidative stress status has a key role in HCC development and progression.

The most important reactive oxygen species (ROS) derived by molecular oxygen include free oxygen radicals [e.g., superoxide (O_2_•-), hydroxyl radical (OH^.^), nitric oxide (NO^.^) radicals] as well as nonradical ROS [e.g., hydrogen peroxide (H_2_O_2_), organic hydroperoxides, and hypochloride].

A low level of ROS is indispensable in several physiologic processes of the cell including proliferation, apoptosis, cell cycle arrest, cell senescence, etc. [85]. However, an increased level of ROS causes oxidative stress and creates a potentially toxic environment to the cells. In normal physiologic condition, a balance between ROS generation and oxidative defences exists in a cell. A significant role is played by endogenous antioxidant enzymes such as superoxide dismutase (SOD), catalase (CAT) that act on O_2_•- and H_2_O_2_, respectively, and glutathione peroxidase (Gpx1) that uses glutathione as co-substrate. Despite the basal production of radicals is hampered by the anti-oxidant defences, the generation of ROS is amplified in response to various environmental perturbations.

This stressful condition is known to play a major role in cancer development mainly by enhancing DNA damage and by modifying some key cellular processes, such as DNA damage caused primarily by hydroxyl radicals [64], cell proliferation, apoptosis, and cell motility cascades by superoxide radicals and hydrogen peroxides playing an important role in cancer development.

Although extensive or limited damage may trigger cell death, many cells can tolerate and repair the occasional hit from ROS. In the Fruehauf model [86], when the balance tips further in favour of ROS, programmed cell death becomes a near certainty.

Excessive ROS, which the cellular enzymes cannot neutralize, alters the chemical environment within the mitochondria; in fact, the pore protein that forms a channel through the mitochondrial membranes becomes jammed in the open position, allowing cytochrome *c *to escape into the cytoplasm thus triggering programmed cell death.

The increase of ROS is associated with the increase of the inducible mitochondrial manganese SOD (MnSOD) expression. Elevated serum MnSOD levels have been found in patients with HCC [87] and relatively high values of the enzyme have also been observed in patients with chronic hepatitis and liver cirrhosis. Therefore, it could be hypothesized that during induction of the malignant process in cirrhotic liver, the increase in MnSOD activity can already occur in the precancerous phase.

In cancer biology, NO can be involved either in promotion or in prevention of tumour occurrence dependently from tumour microenvironment, NO concentration and time of exposure [88]. NO is a product of endothelial cells that binds and activates the guanylate cyclase, which catalyzes the conversion of GTP to the second messenger molecule cyclic GMP (cGMP). Concentrations of NO ranging between 1 and 30 nM produce high levels of cGMP promoting angiogenesis and proliferation of endothelial cells. In these conditions, ERK phosphorylation stimulates the proliferation of endothelial cells. Concentrations of NO ranging between 30 and 100 nM correspond to an increase of proliferative and anti-apoptotic AKT and ERK-dependent pathways in tumour cells [89-91]. This range of concentrations seems to protect tumour cells from apoptosis and enhance angiogenic effects. In these conditions, the molecules activated by NO can be considered as factors correlated to poor prognosis events. On the other hand, higher NO levels (> 300 nM) promote apoptosis and are responsible for anti-tumour activity. NO levels are influenced also by ROS and, specifically, by superoxide anions that can attenuate the NO-mediated pathway. In fact, superoxide anions and ROS, through the scavenging of NO, can lower NO levels favouring its tumour-promoting activity [92]. Accordingly, tumours have high levels of ROS and low levels of SOD.

Similarly to oxidative stress, the expression of nitrosative stress supports the de-regulated synthesis or overproduction of NO and NO-derived products and its toxic physiological consequences [93]. The main source of NO in the mammals is the enzymatic oxidation of L-arginine by NO synthases [94]. As ROS, NO may limit oxidative damage by acting as a chain-breaking radical scavenger or may cause damage and kill cells by mechanisms that include inhibition of protein [95] and DNA [96] synthesis, downregulation of antioxidative enzymes [97] and depletion of intracellular GSH [98]. Nitrosative insult may occur *in vivo *also in pathologies associated with inflammatory processes, neurotoxicity and ischaemia [99].

NO is able to reduce oxidative injury via several mechanisms. NO reacts with peroxy and oxy radicals generated during the process of lipid peroxidation. The reactions between NO and these ROS can terminate lipid peroxidation and protect tissues from ROS-induced injuries [100]. Through the Fenton reaction, hydrogen peroxide oxidizes iron (II) and the process generates an extremely reactive intermediate (the hydroxyl radical) which then carries out oxidations of different substrates [H_2_O_2 _+ Fe^2+ ^→ Fe^3+ ^+ OH- + hydroxyl radical (·OH)]. NO prevents hydroxyl radical formation by blocking the predominant iron catalyst in the Fenton reaction. In fact, NO reacts with iron and forms an iron-nitrosyl complex, inhibiting iron's catalytic functions in the Fenton reaction [101].

Treatment of rat hepatocytes with NO induces resistance to H_2_O_2_-induced cell death by induction of the rate-limiting antioxidant enzyme, heme oxygenase (HO-1) [102]. In addition, NO prevents the induction of some ROS-induced genes during tissue injury such as early growth response-1 (EGR-1), which activates a number of adhesion molecules and accelerates oxidative tissue injuries [103].

Regulatory events and their alterations depend on the magnitude and duration of the change in ROS or RNS concentration. ROS and RNS normally occur in living tissues at relatively low steady-state levels. The increase in superoxide or NO production leads to a temporary imbalance that forms the basis of redox regulation. The persistent production of abnormally large amounts of ROS or RNS, however, may lead to persistent changes in signal transduction and gene expression, which, in turn, may give rise to pathological conditions [104].

### 3.1 Stress and HCC

Oxidative stress has emerged as a key player in both development and progression of many pathological conditions, including HCV- and HBV-induced liver diseases.

ER stress is a homeostatic mechanism, that regulates cellular metabolism and protein synthesis in response to perturbations in protein folding and biosynthesis [105]. Moderate ER stress modulates protein synthesis initiation and causes a reduction in cell growth, whereas extreme or prolonged ER stress leads to apoptosis mediated by the activation of the ER-associated caspase 12 [106].

Signaling from ER susceptible to stress is closely related to cell metabolism and intracellular redox status [107]. Changes in cell metabolism can cause an increase of mutation processes including stimulation of cell proliferation and apoptosis [84].

Studies of mechanisms of oxidative stress have shown that the latter activates signaling cascades (including MAP kinase pathway), which can seriously influence regulation of cell growth and transformation processes [84] and may be involved in pathogenesis of some diseases associated with oxidative stress.

Oxidative stress also activates hepatic stellate cells that represent the main connective tissue cells in the liver, involved in formation of extracellular matrix and required for normal growth and differentiation of cells during liver damage. In this case, the stellate cells divide in response to various cytokines, growth factors, and chemokines produced by the damaged liver. Chronic activation of stellate cells in response to oxidative stress induced by viral replication may contribute to fibrogenesis and increase proliferation of hepatocytes chronically infected with HBV and HCV that, together with activation of MAP kinases, may induce HCC [108].

The nuclear transcription factor-κB (NF-κB) is the major stress-inducible antiapoptotic transcription factor. NF-κB activation is associated with cancer, and it has been found to be strongly activated in many types of cancer, including HCC [108].

Moreover, markers of acute intracellular oxidative stress were found elevated in patients with chronic HCV [109] with accumulation of DNA adduct 8-hydroxydeoxyguanosine [110]. Transgenic mice expressing HCV core protein show an increased accumulation of ROS that correlates with HCC development [111].

The increased generation of ROS and RNS, together with the decreased antioxidant defense, promotes the development and progression of hepatic and extrahepatic complications of HCV infection.

## 4. HCC therapeutic opportunities

Ablative therapies, surgical resection or liver transplantation are the first-line treatment for patients affected by HCC [112,113]. Nonetheless, advanced tumour stage and poor liver function preclude the majority of patients from these surgical interventions [114]. Moreover, transplantation is indicated only for early small HCC, and its application is limited by the availability of liver grafts [115]. Therefore, it is mandatory to develop an effective systemic therapy for patients with advanced HCC.

HCC is a chemo-resistant tumour and conventional cytotoxic chemotherapy has not provided clinical benefit or prolonged survival for patients with advanced HCC [116].

In recent years, emerging insights into the biology and molecular signalling pathways in cancer cells have led to the identification of potential targets for intervention and the advent of promising targeted therapy for the treatment of HCC (Table 1).

**Table 1:** Targeted agents in development for HCC

Agent	Anti-angiogenic targets			Anti-proliferative targets			Clinical Development
	**VEGF**	**VEGFR**	**PDGFR**	**EGFR**	**Raf**	**mTOR**	

**Bevacizumab**	•						Phase II ongoing

**Brivanib**		•					Phase II recruiting

**Cediranib**		•					Phase II recruiting

**Erlotinib**				•			Phase II complete

**Gefitinib**				•			Phase II complete

**Cetuximab**				•			Phase II complete

**Lapatinib**				•			Phase II ongoing

**RAD001**						•	Phase I/II recruiting

Sorafenib*		•	•		•		Phase III complete

Sunitinib*		•	•				Phase II ongoing

**Thalidomide**	•						Phase III recruiting

*Sorafenib and sunitinib are multi-tyrosine kinase inhibitors having both anti-proliferative and anti-angiogenic effects

### 4.1 Erlotinib

With recent advances in the knowledge of hepato-carcinogenesis, there has been encouraging development in the systemic therapy of advanced HCC patients, and particularly in the therapy based on specific targets ("targeted therapy"). Among the newly identified targets, interesting results have been shown in targeting the epidermal growth factor receptor/human epidermal growth factor receptor 1 (EGFR/HER1) and its ligands EGF and transforming growth factor-alpha (TGF-α), important in cell proliferation, as well as motility, adhesion, invasion, survival, and angiogenesis [117,118]. It has been suggested that hypomethylation of the EGF receptor gene may be associated with the development of HCC [119]. Studies have indicated that EGFR/HER1 is actively expressed in human hepatoma [120]. Different phase II studies of Erlotinib (Tarceva, OSI-774; OSI Pharmaceuticals, Melville, NY), an orally active, potent, selective inhibitor of the EGFR/HER1-related tyrosine kinase enzyme were performed in patients with HCC [121,122].

In the study by Philip et al. [121] 3 of 38 patients (9%) achieved partial responses (PR) and 12 of 38 patients (32%) were free of disease progression (PD) at 6 months. In another preliminary report by Thomas et al., [122] 8 of 25 patients (32%) achieved a median progression-free survival (PFS) of 4 months.

### 4.3 Cetuximab

Cetuximab, a chimeric monoclonal Ig G1 antibody directed against the EGFR that blocks binding of endogenous EGFR ligands, was recently evaluated in HCC patients who had previously received 1 or 2 lines of systemic chemotherapy regimens. Cetuximab was well tolerated, and through concentrations only mild to moderate hepatic dysfunction were observed. However, there were no tumor responses, and the median PFS was only 1.4 months [123].

In another trial [124] Cetuximab was combined with Gemcitabine and Oxaliplatin chemotherapy (GEMOX regimen) in patients with documented progressive HCC. The confirmed response rate was 20% and disease stabilization (SD) was obtained in 40% of patients. On the other hand, the toxicity profile was not neglactable (60% of grade 3 or 4 toxicity), although still acceptable.

### 4.4 Bevacizumab

Bevacizumab is a recombinant humanized anti-VEGF monoclonal antibody, thereby inhibiting neo-angiogenesis, tumour growth, paracrine/autocrine growth factor release and metastasis. Bevacizumab, both as a single agent and in combination with other agents, has shown initial encouraging activity in treating advanced HCC. In the study by Siegel et al. [125], among 46 patients enrolled with advanced HCC, single-agent bevacizumab induced a 13% objective response (OR), while 65% of the patients had SD.

Bevacizumab and erlotinib combination was also investigated in advanced or metastatic HCC at phase II trials. This regimen consists of bevacizumab 10 mg/kg every 14 days and erlotinib 150 mg orally daily, continuously, for 28-day cycles. Of 40 patients, 62.5% survived beyond 16 weeks without evidence of progression. Ten patients achieved a PR, while median PFS and overall survival (OS) were 9.1 and 15.9 months, respectively [126].

All these seemingly promising results are mostly based on small, non-randomized phase II studies.

### 4.5 Sunitinib

Another potential promising multitargeting agent is sunitinib, which is an inhibitor of VEGFR, PDGFR-α and β, c-kit, Flt-3 and RET kinases [127].

European/Asian phase II study explored the safety and efficacy of sunitinib dosed at 50 mg daily for 4 weeks in 37 patients with unresectable HCC. Since only one PR was confirmed, with prevalent SD recorded, the trial did not proceed to the second stage. Moreover, Sunitinib showed pronounced toxicities at a dose of 50 mg/day in patients with unresectable HCC. The response rate was low, and the study did not meet the primary endpoint based on RECIST criteria [128].

Different chemotherapy strategies to use in HCC treatment exploit the intrinsic oxidative stress of tumour cells. The first attempt to employ in vivo pro-oxidant agents was reported by Nathan e Chon in 1981 that used the glucose oxidase as H2O2 precursor obtaining a significant decrease of tumour growth [129]. Various chemotherapy agents actually in use, including doxorubicin, vinblastine, vincristine and camptotecin, have a redox H_2_O_2_-mediated activity [130] on tumour cells without effects on health tissues [131].

The main systemic therapy to prolong survival in patients with advanced HCC and the new reference standard for systemic treatment for these patients is sorafenib [132].

### 4.6 Sorafenib

Sorafenib (Nexavar BAY 43-9006) is a multikinase inhibitor that has shown efficacy against a wide variety of tumours in preclinical models and clinical studies.

It has been shown to block tumour cell proliferation and angiogenesis by inhibiting serine⁄ threonine kinases [c-RAF, and mutant and wildtype B-RAF (v-raf murine sarcoma viral oncogene homolog B1)] as well as the receptor tyrosine kinases VEGFR2, VEGFR3, PDGFR, FLT3, RET and c-KIT. On the other hand, it is known that the overexpression and/or mutation of Raf kinase is a common event in several tumours, including HCC. In fact, RAF kinases are key regulators of the MEK⁄ERK cascade and up-regulated signalling through the RAF/MEK/ERK pathway has an important role in HCC [133].

The efficacy of sorafenib on HCC has been confirmed in both phase II and phase III trials [134-136]. In the large randomized phase III Sorafenib HCC Assessment Randomized Protocol (SHARP)-SHARP study, 602 patients with biopsy-proven advanced HCC who had not received any prior systemic treatment were evaluated and randomized to receive either sorafenib (400 mg twice daily, n = 299) or a placebo. The primary endpoints were OS and time to symptomatic progression, while the secondary endpoint was time to progression (TTP). The results demonstrated a significant improvement in both OS (median 10.7 vs 7.9 months) and TTP (median 5.5 vs 2.8 months) in the sorafenib group vs the placebo group. These results indeed represented a 44% increase in OS and 73% prolongation in the TTP.

The SHARP protocol represents the first large-scale randomized trial that demonstrates the OS benefit of systemic treatment in patients with advanced HCC thus far, and therefore it has been approved by the US Food and Drug Administration for the treatment of advanced HCC patients. However, this study was conducted mainly in the western countries, where the main etiologies of HCC are HCV and alcohol. In contrast, the main bulk of HCC occurs in Asian countries, where chronic HBV infection accounts for the majority of HCC cases. Therefore, similar to the design of the SHARP study, an Oriental sorafenib study was performed to investigate the efficacy and tolerability of using single-agent sorafenib in treating advanced HCC patients in Asian population.

In this study, the median OS of patients on sorafenib was 6.2 months, which was significantly better than 4.1 months achieved in patients on placebo.

Although these two pivotal studies have demonstrated good activity and tolerability in treating advanced HCC patients with sorafenib, it is still far from an efficient control of this disease.

The combination of sorafenib with agents active in the control of the HCC-derived symptoms could be useful in the clinical strategy of HCC in order to increase treatment tolerability.

Combination of molecular therapies is expected to improve the outcome benefits obtained with sorafenib, but this is a highly complex matter due to the complexity of complementary pathways activated in HCC. Examples of this are given by the combination of sorafenib with anti-angiogenic agents and blockers of cell proliferation, such as EGFR, MET, and IGFR inhibitors. An alternative strategy is to combine therapies abrogating complementary intracellular signaling, such as RAS or mTOR inhibitors. Similarly, proapoptotic agents might synergize with cell proliferation inhibitors [59,137]

### 4.7 Octreotide

Differential somatostatin receptor subtypes (SSTR 1, 2, 3 and 5) are expressed in HCC [138]. Somatostatin analogues, such as octreotide, which display high binding affinity to SSTR2 and lower affinity to SSTR5 and SSTR3 (affinity rank order: SSTR2 > SSTR5 > SSTR3) are efficacious in the treatment of neuroendocrine tumors and exhibit only mild toxicity [139].

Octreotide LAR (long-acting release) is a formulation of octreotide encapsulated into microspheres of the biodegradable glucose star polymer [140]. This synthetic version of somatostatin differs from the latter for the prolonged half-life that allows to administer the drug every 28 days to obtain active plasma concentrations.

As somatostatin, octreotide reduces the release of growth factors and inhibits neo-angiogenesis. Octreotide was previously used in HCC patients with conflicting results [140,141]. However, approximately 40% of patients respond to octreotide with improved survival and an impressive quality of life [142]. We showed, in a previous study, that combination of octreotide and radiofrequency ablation produced about 80% of disease control and interesting mean OS (31.4 months) in a series of advanced HCC patients [143]. Investigations on octreotide in HCC are still ongoing also as National Cancer Institute sponsored trials [144,145].

Based on these premises, our group started a phase II multicenter study based on the combination between sorafenib and octreotide LAR (So.LAR protocol) in order to assess its safety and activity in advanced HCC patients [146]. Five PR (10%), 33 SD (66%) and 12 PD (24%) were recorded. Overall disease control rate (CR+PR+SD) was 76%. In conclusion, the combination between sorafenib and octreotide LAR was active and well tolerated in advanced HCC [139]. Moreover, we have investigated on the pharmaco-dynamic interference between the two agents and the level of Erk activation that serves as a surrogate of the inhibition induced by Sorafenib [147]. In details, we have evaluated the effects of So.LAR treatment on Erk activity in PBMC of patients affected by HCC with cytofluorimetric technique. We found a gradual reduction of Erk1/2 activity in 15 patients responsive to the treatment reaching an about 50% maximal decrease after 21 days (T21) from the beginning of therapy. On the other hand, in 17 patients resistant to treatment the activity of Erk1/2 was about 80% increased at T21. An opposite trend of intracellular O^2- ^levels was observed in resistant patients. These effects were correlated to the modulation of SOD activity (physiological scavenger of O^2-^) and of serum NO levels. In fact, in 20 responder patients, sorafenib alone induced an increase of about 40% of NO levels and of about 2-fold of SOD activity and this latter effect was significantly potentiated after the addition of octreotide LAR. In conclusion, the determination of both pErk expression in PBMC and the oxidative stress status have high value in the prediction of response to sorafenib+octreotide therapy in HCC patients.

The increased generation of acute intracellular oxidative stress, which results from the generation of reactive oxygen species (ROS) by environmental factors or cellular mitochondrial alterations, has recently been associated with the progression of chronic liver diseases and hepatocarcinogenesis. On the other hand, a distinctive pathological hallmark of HCC is a dramatic down-regulation of oxidoreductase enzymes that constitute the most important free radical scavenger systems represented by catalase, SOD and glutathione peroxidase [87,148-150].

## 5. Biomarkers of oxidative stress

One strong mechanistic link between chronic inflammation and cancer is through the increased production of free radicals at the site of inflammation and the resulting molecular changes, which include lipid peroxidation and oxidative DNA damage [151]. Indeed, markers of DNA damage, such as 8-hydroxydeoxyguanosine (8-OHdG), and lipid peroxidation, such as 4-hydroxynonenal (HNE) and malondialdehyde (MDA), are commonly elevated in liver of patients with chronic HCV infection and correlate well with the degree of viral infection and inflammation, known risk factors for HCC [152].

In addition to the classical genetic mechanisms of deletion or inactivating point mutations, epigenetic alterations, such as hyperacetylation of the chromatin-associated histones are believed to be involved in the development and progression of HCC. Histone deacetylases (HDACs) are important regulators of many oxidative stress pathways including those involved with both sensing and coordinating the cellular response to oxidative stress. In particular aberrant regulation of these pathways by HDACs may play critical roles in cancer progression. Infact, HA-But, an HDAC inhibitor in which butyric acid residues are esterified to a hyaluronic acid backbone and characterized by a high affinity for the membrane receptor CD44, valproic acid and ITF2357, exhibiting inherent therapeutic activity against HCC may represent a promising approach for HCC treatment [153,154].

It is well known that inflammation is one of the biological responses driven by oxidative stress. Modulation of oxidative damage as well as inflammation protect against hepatocarcinogenesis. It has been shown that resveratrol, a compound present in grapes and red wine, has potent antioxidant [155] and anti-inflammatory [156] properties, which might play an important role in protecting the liver against carcinogen-induced neoplasia. Recently, it was reported that resveratrol significantly prevents diethylnitrosamine (DENA)-induced liver tumorigenesis in rats [157].

## Conclusions

HCC is a disease that presents two relevant concerns: i) the presence of a cirrhotic background that severely affects both the quality of life and the survival of the patients and ii) the pleiotropic pathogenesis that has as common background: the chronic inflammation and the oxidative stress. The pharmacological weapons against HCC are still limited and efficacy has been established only for the multiple kinase inhibitor sorafenib. We have recently demonstrated that sorafenib plus octreotide is a safe and effective option in advanced HCC patients with compromised metabolic scores and/or low performance status. A still limited covered area of research in HCC is represented by the oxidative stress that underlies primary liver tumour development and that occurs through the generation of ROS and/or RNS and that is regulated by several scavenger mechanisms. On this view, we have found that the determination of oxidative stress status has high value in the prediction of response to sorafenib plus octreotide therapy in HCC patients. These data could have a profound impact in the determination of the sensitivity of the patients to this pharmacological strategy and could have a role in the selection of the patients to be subjected to this treatment. This could reduce both the relevant side effects correlated to the therapy and the relevant costs derived from the use of expensive drugs such as the new target based agents such as sorafenib. The factors involved in the oxidative stress could have a role not only in the prediction of response to pharmacological treatments but could be themselves targets of drugs as in the case of the stress-dependent kinases p38 kinase and Jun kinase or in the case of the use of anti-oxidant agents such as resveratrol or silibin. The investigations on oxidative stress and on its connection with signal transduction pathways correlated to survival and/or proliferation could disclose new scenarios of interventions based on the rational use of anti-oxidant agents in combination with target based drugs.

## Competing interests

The authors declare that they have no competing interests.

## Authors' contributions

MM, MC, ML, AL, AA and PS have critically revised the manuscript and have made substantial contributions to conception MA, IMS, LT, AG, MA, PS and RS have been involved in drafting the manuscript. MC, MM and PS have given final approval of the version to be published. All authors have read and approved the final manuscript.
